# Estimation and probabilistic projection of age- and sex-specific mortality rates across Brazilian municipalities between 2010 and 2030

**DOI:** 10.1186/s12963-024-00329-x

**Published:** 2024-05-27

**Authors:** Marcos R. Gonzaga, Bernardo L. Queiroz, Flávio H.M.A. Freire, José H.C. Monteiro-da-Silva, Everton E.C. Lima, Walter P. Silva-Júnior, Victor H. D. Diógenes, Renzo Flores-Ortiz, Lilia C. C. da Costa, Elzo P. Pinto-Junior, Maria Yury Ichihara, Camila S. S. Teixeira, Flávia J. O. Alves, Aline S. Rocha, Andrêa J. F. Ferreira, Maurício L. Barreto, Srinivasa Vittal Katikireddi, Ruth Dundas, Alastair H. Leyland

**Affiliations:** 1 Graduate Program in Demography, Universidade Federal do Rio Grande do Norte (UFRN), Natal, Rio Grande do Norte Brazil; 2 Graduate Program in Demography, Universidade Federal de Minas Gerais (UFMG), Belo Horizonte, Minas Gerais Brazil; 3 Graduate Group in Demography, University of Pennsylvania, Philadelphia, PA USA; 4 Graduate Program in Demography, Universidade Estadual de Campinas (UNICAMP), Campinas, São Paulo, Brazil; 5 Universidade Federal da Bahia (UFBA), Salvador, Bahia Brazil; 6 Centro de Integração de Dados e Conhecimentos para a Saúde (Center of Data and Knowledge Integration for Health) - CIDACS/ Gonçalo Moniz Institute - Fiocruz/Bahia, Salvador, Brazil; 7 School of Nutrition, Universidade Federal da Bahia (UFBA), Salvador, Brazil; 8 MRC/CSO Social and Public Health Sciences, Unit University of Glasgow, Glasgow, Scotland

**Keywords:** Mortality, Life expectancy, Lee-Carter, population forecast, Small area analysis

## Abstract

**Background:**

Mortality rate estimation in small areas can be difficult due the low number of events/exposure (i.e. stochastic error). If the death records are not completed, it adds a systematic uncertainty on the mortality estimates. Previous studies in Brazil have combined demographic and statistical methods to partially overcome these issues. We estimated age- and sex-specific mortality rates for all 5,565 Brazilian municipalities in 2010 and forecasted probabilistic mortality rates and life expectancy between 2010 and 2030.

**Methods:**

We used a combination of the Tool for Projecting Age-Specific Rates Using Linear Splines (TOPALS), Bayesian Model, Spatial Smoothing Model and an ad-hoc procedure to estimate age- and sex-specific mortality rates for all Brazilian municipalities for 2010. Then we adapted the Lee-Carter model to forecast mortality rates by age and sex in all municipalities between 2010 and 2030.

**Results:**

The adjusted sex- and age-specific mortality rates for all Brazilian municipalities in 2010 reveal a distinct regional pattern, showcasing a decrease in life expectancy in less socioeconomically developed municipalities when compared to estimates without adjustments. The forecasted mortality rates indicate varying regional improvements, leading to a convergence in life expectancy at birth among small areas in Brazil. Consequently, a reduction in the variability of age at death across Brazil’s municipalities was observed, with a persistent sex differential.

**Conclusion:**

Mortality rates at a small-area level were successfully estimated and forecasted, with associated uncertainty estimates also generated for future life tables. Our approach could be applied across countries with data quality issues to improve public policy planning.

**Supplementary Information:**

The online version contains supplementary material available at 10.1186/s12963-024-00329-x.

## Background

Accurate estimation and projection of age-specific mortality rates in small areas are crucial for public health, economic, and social planning [1, 2]. These rates can also be used to monitor the Sustainable Development Goals and aid in the development of other small area indicators [3–5]. However, estimating mortality rates in small areas is still challenging for demographers and epidemiologists in low- and middle-income countries, including Brazil [6–10]. These challenges are due to (1) a limited number of events and exposure in small areas which add stochastic error on the estimates [11–17], and (2) low levels of completeness of death registrations, which may be related to under-reporting of death counts in vital records, a kind of systematic error particularly common in developing countries [18–22].

There is considerable research on small area mortality rate estimation in developed countries, especially those with reliable vital records [23–25]. However, in Brazil, mortality rates are affected due to the incompleteness of vital records [17, 19, 21]. Despite significant improvements in vital register quality at the national level in the last few decades, the lack of complete death records remains a considerable challenge in most of Brazil’s less developed areas, particularly the north and northeast macro-regions [21, 26].

In the broader context, the territorial configuration of the Unified Health System (Sistema Único de Saúde – SUS, in Portuguese) in Brazil reflects and perpetuates regional disparities [27]. During the initial decade of its implementation (1990–2000), the spatial distribution of public health services mirrored the trends of decentralization and inequality that were present in the country [28]. Specialized medical facilities of medium and high complexity remained primarily concentrated in capital cities, metropolises, and a few regional hubs [27]. This concentration resulted in substantial disparities in patient flows, with higher patient numbers for more complex medical services [29]. In contrast, primary care, especially the teams associated with the Family Health Program (Programa de Saúde da Família – PSF, in Portuguese), experienced a different trajectory. There was a notable expansion of Family Health in the economically disadvantaged regions of the country (especially in the North and Northeast of Brazil), with challenges in its implementation in the peripheries of metropolitan areas, which were typically the wealthiest and most densely populated regions [30]. This distribution of healthcare resources significantly impacted the social and geographic inequalities in access to healthcare services, resulting in marked disparities between residents of economically developed (Southern and Southeastern parts of Brazil) and less developed Northern and Northeastern regions [29, 31]. These spatial inequalities in health services access are also reflected in an uneven regional quality of death records.

Brazilian studies on mortality in small areas suggest a combination of methods to address both the stochastic and systematic error sources. The standard approach is the use of demographic death distribution methods to estimate the degree of completeness of death count registrations of adults across macro-regions or states [32–36], combined with direct/indirect standardization techniques [37] to smooth and estimate age-specific mortality rates in small areas [19, 38]. These approaches are limited for three main reasons. First, all small areas will have the same mortality structure as the standard. Second, there is an importance of selecting a suitable mortality pattern. Finally, the uncertainty quantification of the direct/indirect standardization does not consider all the relevant sources of errors: stochastic errors, errors in parameter estimates and errors in the completeness of death counts [17], and thus gives an optimistic picture of the uncertainty around age specific mortality estimates. This issue is more pronounced when analyzing sub-national groups [11, 39].

Schmertmann and Gonzaga [17] recently developed a Bayesian regression model that addresses the three limitations above. The method combines a relational model for mortality schedules with probabilistic prior information on death registration coverage, based on several sources of information or public health/demography experts’ opinions.

This study produces small area estimates of age-specific mortality rates by sex and forecasts mortality for these areas until 2030. To summarize, the paper has three main goals. Firstly, we applied the TOPALS relational model proposed by Gonzaga and Schmertmann [14] to smooth and estimate sex- and age-specific mortality rates for Brazilian microregions (a group of municipalities) and municipalities in 2010, without any adjustment for under-registered deaths. Secondly, we applied the Bayesian model proposed by Schmertmann and Gonzaga [17] to estimate microregion-specific mortality rates, accounting for under-registered deaths. Subsequently, we utilized the estimated microregion rates, both adjusted and unadjusted for under-registered deaths, to compute sex- and age-specific completeness estimates of deaths. We then assumed homogeneous death under-registration across all municipalities within a microregion and adjusted municipal rates using the completeness estimates derived from encompassing microregions. Thirdly, we forecasted sex- and age-specific mortality rates, and life tables at the municipal level to 2030, using the Lee-Carter model. We argue that the combination of such methods can be flexible enough to be applied to other developing countries with vital registration problems like Brazil.

This study contributes with two replications and one innovation. The replication of the methods suggested by Schmertmann and Gonzaga [14, 17] into an updated mortality database allows to produce complete sex- and age-specific mortality rates from age 0 to 99 years old in all 5,565 municipalities of Brazil in 2010, simultaneously adjusting for the low number of events/exposure, and incomplete coverage of vital records. The innovation was the probabilistic forecast of mortality rates from 2010 to 2030, adding essential and valuable information for local public health planning. In the context of a pandemic, such as COVID-19, small area mortality estimates and projections are critical to investigate and understand the impact of a new disease [40, 41].

## Data and methods

We used death counts by sex, age, and municipality from the Mortality Information System (data are available at https://datasus.saude.gov.br*).* Population counts by sex, age, and municipality were taken from the 2010 Brazilian Census (data are publicly available at https://www.ibge.gov.br). The country’s five macroregions were subdivided into states (27 total); the states were subdivided into mesoregions (137 total), mesoregions into microregions (558), and microregions into municipalities (5,565). Municipalities are the smallest areas responsible for registering vital events. Additional information on death registration coverage estimates was obtained from the following research projects:

1. Field audit death search in the Northeast and Legal Amazon [18, 42];
2. Research documentation and results provided by Schmertmann and Gonzaga [14, 17]: R codes and results are available at http://topals-mortality.schmert.net/ and http://mortality-subregistration.schmert.net/, respectively.
3. Research Project: “Estimates of Mortality and Table Construction for Small Areas in Brazil, 1980–2010” (process numbers: 470,866/2014 and 454,223/2014-5). Process and results are available at Queiroz et al. [38].
4. Other official estimates and research papers/dissertations on estimates of death count coverage and mortality rates at different geographical levels [21, 33–48].

### Estimating and forecasting sex- and age-specific mortality rates

**Fig. 1:**
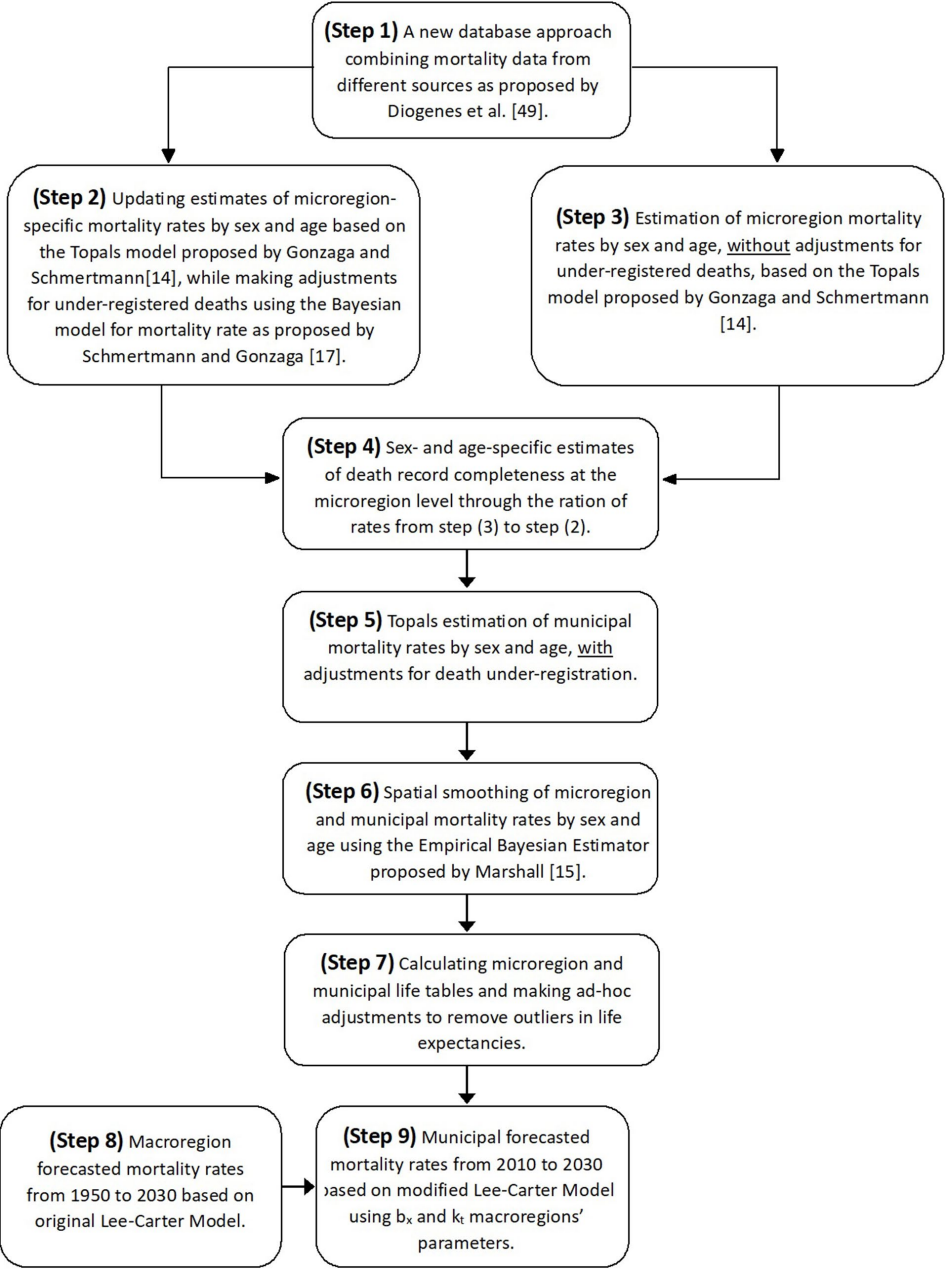
Representation of the modeling strategy for estimating and forecasting age- and sex-specific mortality rates for all municipalities in Brazil Source: Prepared by the authors

We combined statistical and demographic models to estimate and project age-specific mortality rates for all microregions and municipalities of Brazil between 2010 and 2030. Our methodological approach comprises nine steps, as outlined in the flowchart depicted in Fig. 1. Initially, we combined mortality data from the Mortality Information System (SIM) and the 2010 Brazilian Census to establish a new database featuring the most comprehensive death counts. These counts were categorized by sex, age, and municipality, aligning with the methodology proposed by Diogenes et al. [49]. In the second step, we use this newly constructed database of death counts to employ the Bayesian model [17] and revise mortality estimates for all 558 Brazilian microregions in 2010, while considering undercounted deaths counts.

As argued by Schmertmann and Gonzaga [17], any statistical model for mortality rates with incomplete death registrations is susceptible to identifiability issues in mortality rate estimation. It occurs since one cannot distinguish between high mortality/low registration and low mortality/high registration situations. On the other hand, a Bayesian approach allows us to use probabilistic information on which death coverage probabilities are more likely (expressed as a prior distribution) to produce probabilistic statements on mortality rates, given death completeness estimates and exposures. We used a Bayesian model that allows estimation of sex- and age-specific mortality rates in Brazilian microregions, assuming incomplete death registrations. This estimation depends on two types of prior information: sex- and age-specific mortality schedules and the likelihood levels and age patterns of death under-registration, which can be estimated with demographic [32–36] or statistical methods [50]. Unfortunately, it is not possible to obtain reliable information on the age pattern of completeness of vital registration records at the municipal level in Brazil, which is necessary as prior information, to apply the Bayesian model directly to the municipal level.

Our solution, outlined in the subsequent steps of Fig. 1, involved first employing the TOPALS model [14] to estimate and smooth mortality rates by sex and age in the microregions without adjustments for under-registered deaths (step 3). TOPALS is a relational model that represents the sum of two functions [14]: (1) mortality rates representing a basic pattern by age and sex, and (2) a linear parametric function composed of line segments between designated ages (knots), which represents the differences between the mortality function used as a standard and the logarithm of the mortality function of the population of interest. The vector of logarithmic mortality rates of the population of interest is given by:

1 $$\lambda \left(\alpha \right)={\lambda }^{*}+ B \alpha$$

where λ is a 100 × 1 vector of log mortality rates in a microregion, λ* is a standard schedule (the national log mortality rate), B is a matrix of constants in which each column is a linear B-splines basis function with knots defined at exact ages (x = 0, 1, 10, 20, 40, 70, 100), and α is a vector of parameters representing offsets to the standard schedule. In Eq. (1), following Gonzaga and Schmertmann [14], the α values represent additive offsets (λ - λ*) to the log mortality rate schedule at knots between which the offsets change linearly with age.

For any specific set of death and population data by age {*D*_*x*_, *N*_*x*_}_*x*=0…99_, the TOPALS method assumes that deaths are distributed as independent Poisson variables with a log-likelihood:

2 $$\text{Log}L\left(\alpha \right)=constant+ \sum _{x}\left[{D}_{x}{\lambda }_{x}\left(\alpha \right)-{N}_{x}\text{exp}\left({\lambda }_{x}\left(\alpha \right)\right)\right]$$

The sum on the right-hand side of Eq. (2) is a penalty term aimed at preventing any fitted function from being implausible regarding the typical behavior of the logarithm of mortality rates in human populations.

TOPALS model is also utilized in the Bayesian model framework to provide a stable prior for estimating mortality rates in very small populations [17]. Priors for completeness of death registration comes from published estimates based on Death Distribution Methods [21] and field audit project [18, 42]. Then, based on age-specific exposure (N) and registered deaths (R), the model combines prior distributions for coverage (f_𝜋_) and mortality parameters (f_𝛼_) with the Poisson likelihood to produce a posterior marginal distribution for adjusted microregion mortality rates:

3 $$P\left(\alpha |R, N\right)=\int L\left(R|N, \alpha,\pi \right){ f}_{\alpha }\left(\alpha \right){ f}_{\pi }\left(\pi \right) d\pi$$

Subsequently, in step 4, we computed the level of death registration coverage in the microregions by sex and age. This was achieved by dividing the unadjusted mortality rates (step 3) by the adjusted rates (step 2).

In step 5, we adjusted the municipal rates, as estimated in step 3, using the completeness estimates from the enveloping microregions. This approach required assuming that death under-registration is homogeneous across all municipalities within a microregion. Most Brazilian municipalities have small population sizes, leading to potential random variations in the estimates. Conversely, considering a microregion as a cluster of neighboring municipalities with similar regional, demographic, or socioeconomic characteristics - and potentially shared healthcare facilities - we consider this as a reasonable assumption, which may also be employed in other studies [19, 38].

We constructed life tables for all 5,565 Brazilian municipalities in 2010 based on the estimated and adjusted municipal mortality rates. However, in certain municipalities, life expectancy at birth exceeded expectations based on the municipality’s socioeconomic development level. Therefore, in step 6, aiming to mitigate outliers in life expectancy, we opted to employ a spatial smoothing procedure on mortality rates, utilizing the Empirical Bayesian Estimator proposed by Marshall [15]. However, despite these efforts, some estimates remained exceptionally high in certain municipalities. Our ultimate approach (step 7) involved implementing an ad-hoc procedure for life expectancy that surpasses the third quartile plus 1.5 times the interquartile range (Q3 + 1.5IQR). We chose to adopt the microregion mortality rates for municipalities where the life expectancy exceeded Q3 + 1.5IQR in the distribution. Fortunately, out of the 5,565 municipalities, only 7% (382) required adjustment via an ad-hoc procedure (n = 35 in the North, n = 95 in the Northeast, n = 83 in the Southeast, n = 125 in the South, and n = 44 in the Midwest macroregion).

Due to the subsequent procedures from steps 4 to 7, we could not provide Bayesian credible intervals for municipal mortality rates. Thus we decide to consider the 2010 municipal mortality rates as the true rates. That could be a very strong assumption since, as we highlighted earlier, mortality rates in small areas could be affected by more than one types of error. However, median or point estimates could be essential and valuable information for local public health planning.

Finally, in steps 8 and 9 we applied the Lee-Carter model to forecast mortality rates by age and sex in all municipalities between 2010 and 2030. The Lee-Carter model [51] combines a demographic model with a time-series method, since it involves modeling two factors: age and time and uses matrix decomposition to extract a single time-varying index of mortality rates, which is then forecasted using a time-series model. The Lee-Carter model is considered a powerful method to forecast mortality rates, due to its precision and simple way of modeling the age distribution of death rates [52–54]. There are variations of the Lee-Carter model, but we used an adaptation of the original one [51] since it was more efficient in dealing with our data limitations.

To estimate the Lee-Carter model parameters, we needed a matrix of mortality rates by age, and a solution for the following equation:

4 $${\rm{log}}\left({{m_{x,t}}} \right) = {a_x} + {b_x}{k_t} + {\epsilon _{x,t}}$$

where *m*_*x, t*_ is the mortality rate, *a*_*x*_, *b*_*x*_, and *k*_*t*_ are the parameters to be estimated, and *ε*_*x, t*_ is a set of random disturbances. The *x* and *t* represent age and year, respectively. The solution to this equation is achieved by applying the Singular Value-Decomposition approach (SVD) to the historical mortality rates matrix log. In the model, *a*_*x*_ represents the average age pattern of the mortality; *b*_*x*_ represents the amount of mortality change at a given age for a unit of yearly mortality change, and *k*_*t*_ measures the general level of mortality over time.

We have municipal adjusted mortality rate estimates only for 2010 and not time series estimates. Our solution was first to apply the Lee-Carter model to a long time-series of mortality rates for the five macroregions of Brazil (South, Southeast, Central-West, North, and Northeast) between 1950 and 2010, based on data available in Silva [47], as described in step 8 of Fig. 1. Then, in step 9, we used the larger regions, for which we estimated the Lee-Carter parameters, to forecast mortality rates for all municipalities within each of these areas. We set parameter *a*_*x*_ as the true adjusted age-specific mortality rate estimated for each municipality in 2010 (as estimated in steps 2 to 7). Parameters *b*_*x*_ and *k*_*t*_, for each municipality within a larger region, come from the adjusted model for the larger region. The assumption used here is a convergence between levels and age patterns of log mortality rates for all municipalities within each macroregion. The limitation of this assumption is that the model parameters may be overly influenced by the larger municipalities within each macroregion. On the other hand, under-registration of death counts across Brazil’s macroregions is steadily decreasing, and the hypothesis that the levels of mortality will become more homogeneous across municipalities is plausible [55, 56]. In 1940, the difference between the lowest (Northeast region 36.68) and the highest life expectancy at birth in Brazil (South region 49.19) was 12.51 years. By 2010, this difference had decreased to 5.08 years between the North (70.79) and South (75.87) regions [57, 58].

With this methodology, we obtained adjusted estimates of age- and sex-specific mortality rates for all Brazilian municipalities for 2010. Subsequently, we forecasted mortality rates up to 2030. The estimates and projections are adjusted for two types of uncertainty: the first arising from potential under-reporting of deaths, and the second being stochastic uncertainty.

### Summarizing trends on life expectancy

To summarize the results of forecast analysis, we calculate some measures that allow looking at two types of variability: (1) Variability in life expectancy between regions, and (2) Variability in the life table age at death within a region. First, we calculate the Interquartile Range (IQR) for municipalities life tables death distribution by sex in 2010 and 2030. The Interquartile Range (IQR) is widely used to measure variability in the distribution of mortality across ages. We calculated this measure as the difference between the first and third quartiles of the life table survivor function (lx). The lower the IQR, the less variability in death distribution by age. Then, we calculate the Coefficient of Variation (CV) for life expectancy at birth between the municipalities by sex, macroregion, and year between 2010 and 2030. The CV is a useful measure to compare variability between different populations since it is a standardized measure of dispersion in any frequency distribution. We calculated CV by dividing the standard deviation to the mean of municipalities’ life expectancy.

## Results

**Fig. 2:**
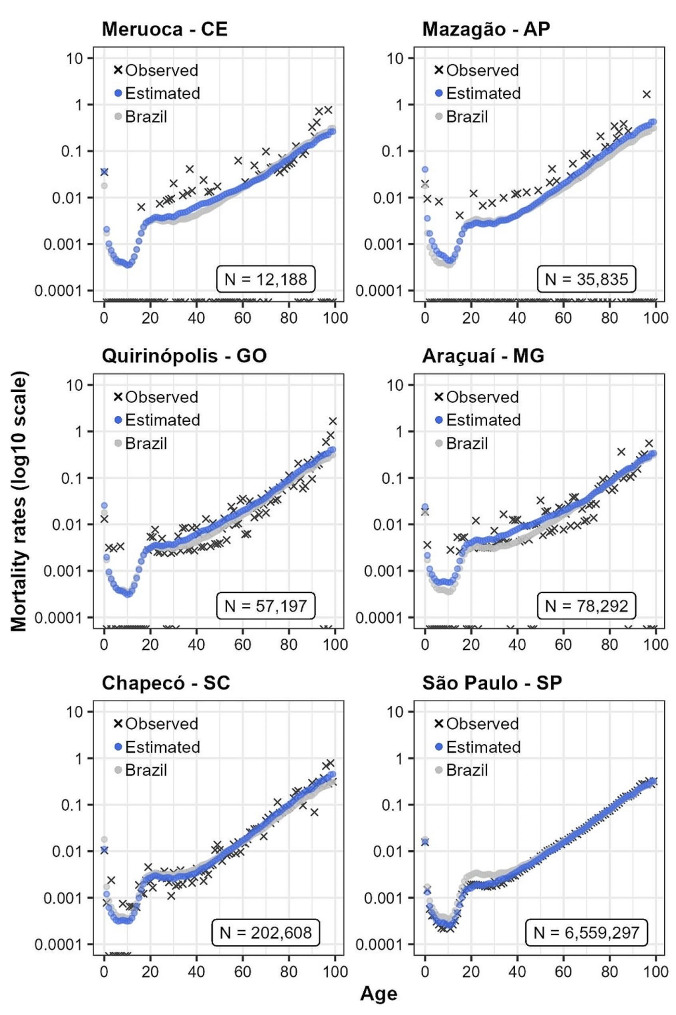
Age-specific mortality rates (log scale) for men in selected municipalities Brazil (2010) Source: Mortality Information System/Ministry of Health (SIM/Datasus) and Brazilian Demographic Census (IBGE, 2010)

Figure 2 shows the logarithm of observed and estimated male age-specific mortality rates in selected municipalities across different macroregions of Brazil for 2010. Municipalities were selected according to different population sizes (N). The results indicate the robustness of combining TOPALS, Bayesian model and spatial smoothing to estimate sex- and age-specific mortality rates for large and small areas. Overall, mortality increases with age and an excess of mortality is also observed for young adults.

Figure 2 highlights the higher variability on males’ mortality risk is associated with lower exposure in the estimated rates. Despite the high variability in the observed rates for the smallest municipalities, estimated rates are somewhat higher than those observed in the first age, except for Chapecó-SC, Meruoca-CE and São Paulo-SP. In other words, in addition to estimating and smoothing rates by age, our approach also adjusts the estimated rates, correcting for the under-registration of death records. In São Paulo, the largest municipality in Brazil, where death records were almost complete in 2010 [18, 26, 42], the estimated rates are very close to those observed. In fact, according to Figure S1 (see supplementary information), the completeness of death estimates in almost all small areas in the South and some areas in the Southeast or Midwest macroregions have complete death records (completeness of death records estimates close to 1) and, therefore, the only source of uncertainty in the estimated mortality rates in these areas is due to the small number of events. In the South macroregion, the completeness of death estimates is highly concentrated somewhat at value 1, which means they have 100% of deaths registered, independent of sex or age interval.

Figure S2 (supplementary information) shows the evolution of probabilistic life expectancy at birth for the same selected municipalities showed in Fig. 1. The evolution of mortality rates at the global level in each municipality depends on the estimates and forecasting of two microregion’s Lee-Carter parameters (i.e., kt and bx), the uncertainty level in the life tables of any forecasted municipality propagates the uncertainty from the macroregions. That could be an explanation for the fact that the level of uncertainty in forecasted life expectancy is not entirely associated with the size of the population. For example, in Figure S2, male life expectancy in Chapecó-SC (with an exposure size of 202,608 in 2010) shows lower uncertainty than male life expectancy in São Paulo-SP (despite having a much larger exposure in 2010.

**Fig. 3:**
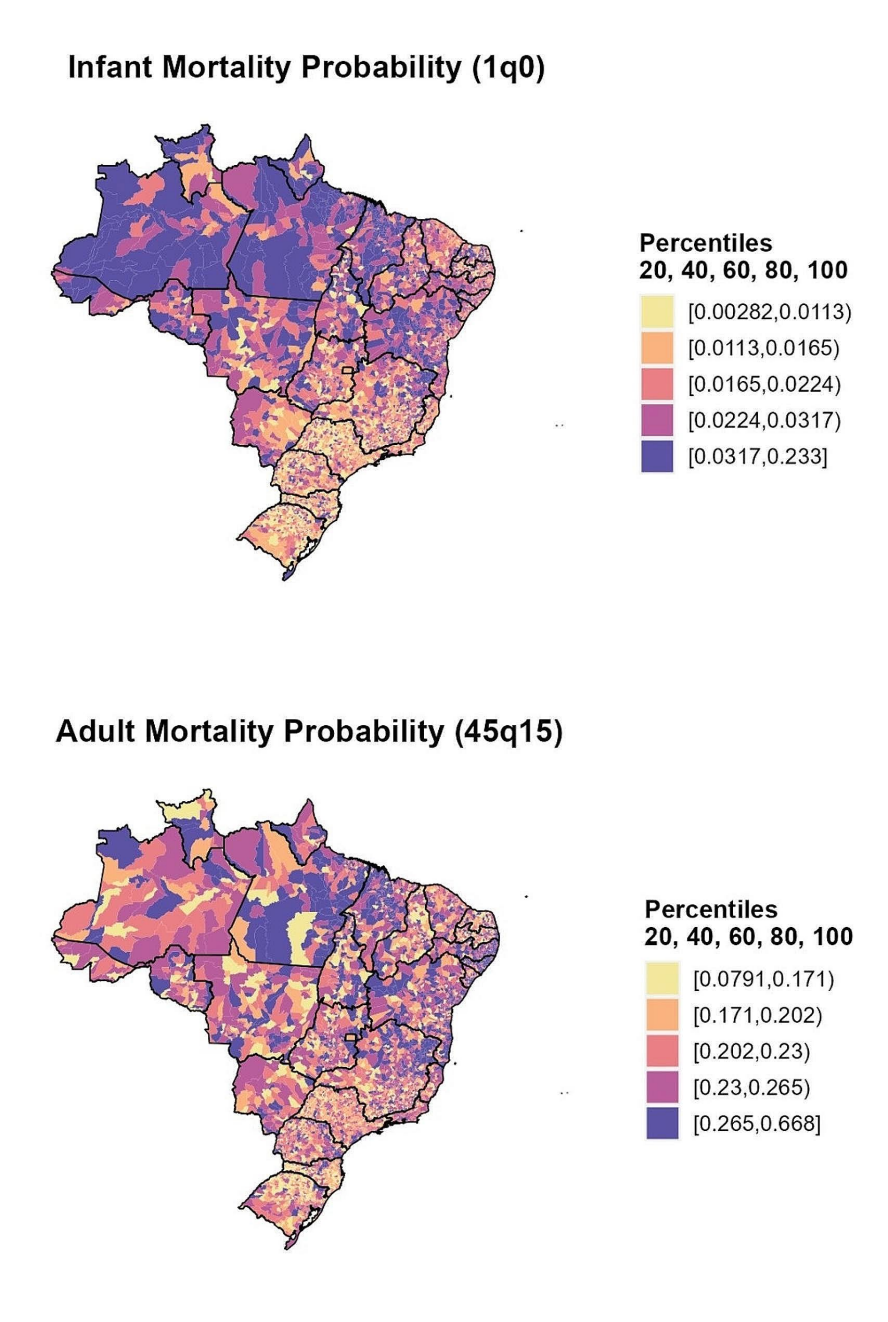
Estimates of male adult and infant mortality rates for the municipalities, Brazil (2010) Source: Mortality Information System/Ministry of Health (SIM/Datasus) and Brazilian Demographic Census (IBGE, 2010)

Figure 3 shows the spatial distribution of infant (_1_q_0_) and adult (_45_q_15_) male mortality probabilities for Brazilian municipalities. Both mortality indicators show the heterogeneity in mortality risk across macro-regions, as observed by others [26, 56, 59, 60]. The lower map shows a higher adult mortality risk in the proximity of the state capitals, especially in the northeast macroregion. The first map shows a concentration of the highest infant mortality levels across the north and northeast macro-regions.

**Fig. 4:**
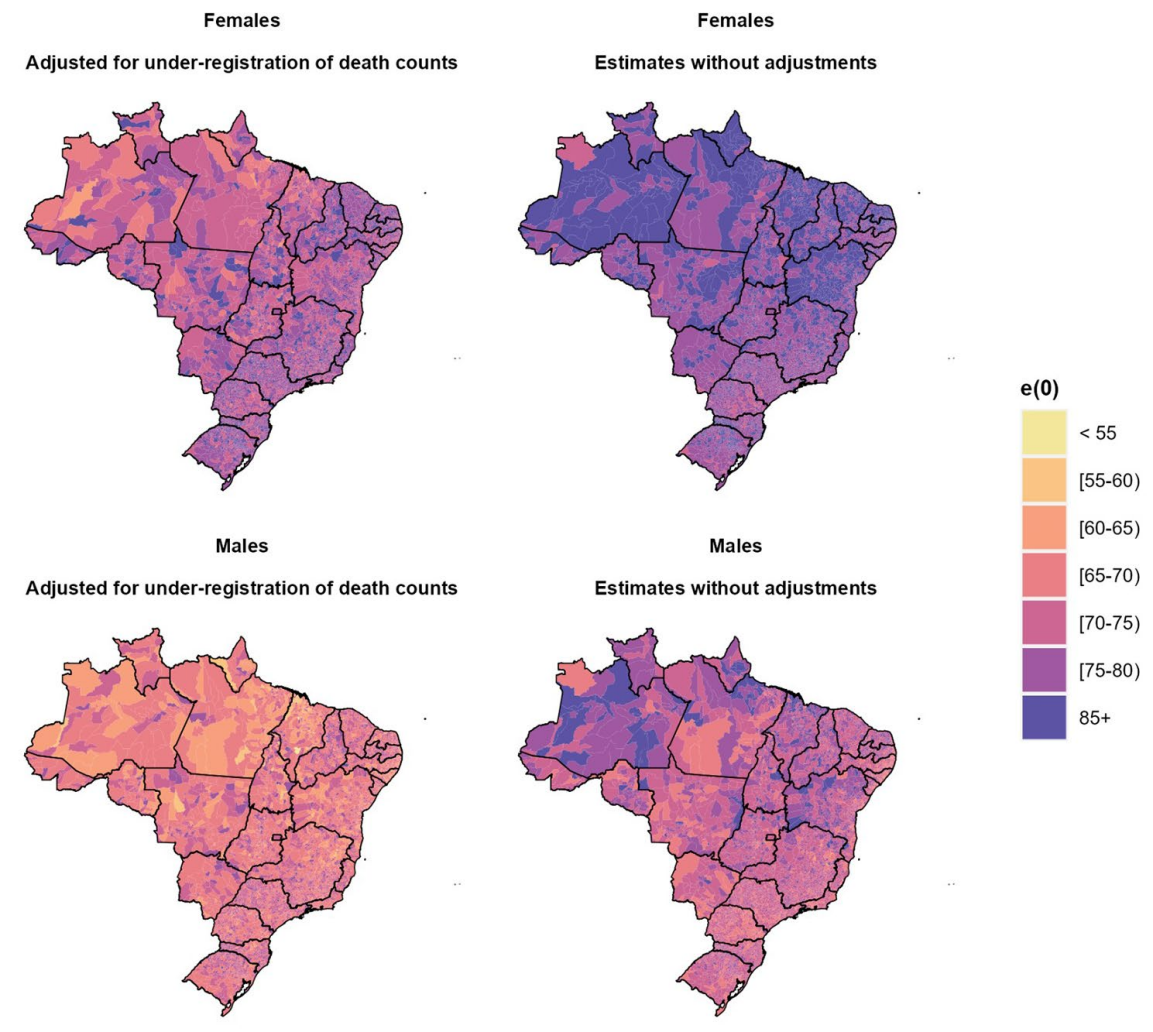
Life expectancy at birth e(0) for the municipalities, Brazil (2010) Source: Mortality Information System/Ministry of Health (SIM/Datasus) and Brazilian Demographic Census (IBGE, 2010)

Figure 4 depicts our estimates and those obtained from SIM on life expectancy at birth e(0) by sex in Brazilian municipalities. The maps displayed on the right show the e(0) estimates without adjustment for completeness of data quality, while those on the left present the estimates using our approach. For instance, without adjustment, we observed very high life expectancy at birth in areas with very high infant and adult mortality, but after adjusting for data quality, the regional distribution was almost the opposite, with the South and Southeast macroregions showing higher levels of e(0). The effect was more evident in the north and northeast macroregions, where a major change in e(0) levels was observed if we compare the highest and lowest percentiles.

**Fig. 5:**
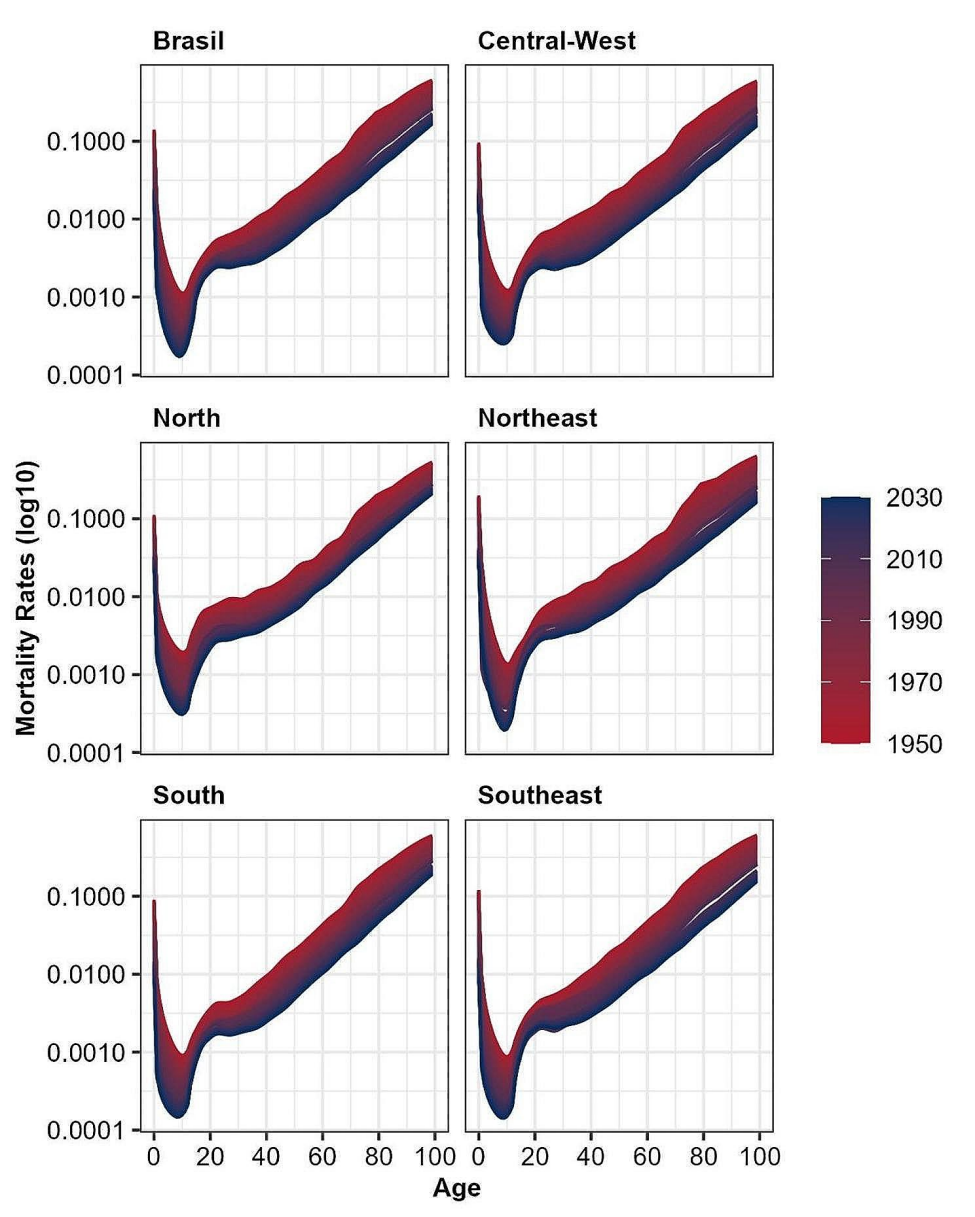
Age-specific mortality rates (log scale) for men in the macro-regions of Brazil (1950–2050) Source: Mortality Information System/Ministry of Health (SIM/Datasus), Brazilian Demographic Census (IBGE, 2010) and Silva [47]

Figure 5 shows observed and forecasted male mortality rates by macroregions of Brazil between 1950 and 2050. Despite still excessive mortality among young adult men, our forecasts suggest a steady decline in male mortality, and increase in life expectancy in all five Brazilian macroregions. For females (supplementary results), there is also a steady decline in mortality in all macroregions. In the national and some regional estimates, we observe a discontinuity in mortality within certain age intervals between 2010 (the last year of past estimates) and 2011 (the first year of forecasted rates). That discontinuity may have resulted from the adjustment and interpolation of rates from 1950 to 2010 using several procedures, according to Silva [47], which could have influenced some Lee-Carter parameters. The author used a combination of model life table methods to obtain mortality estimates and for older ages the analysis employed Gompertz law of mortality to extrapolate mortality above age 70. Unfortunately, we did not have access to the original data to replicate the estimates and test alternatives.

**Fig. 6:**
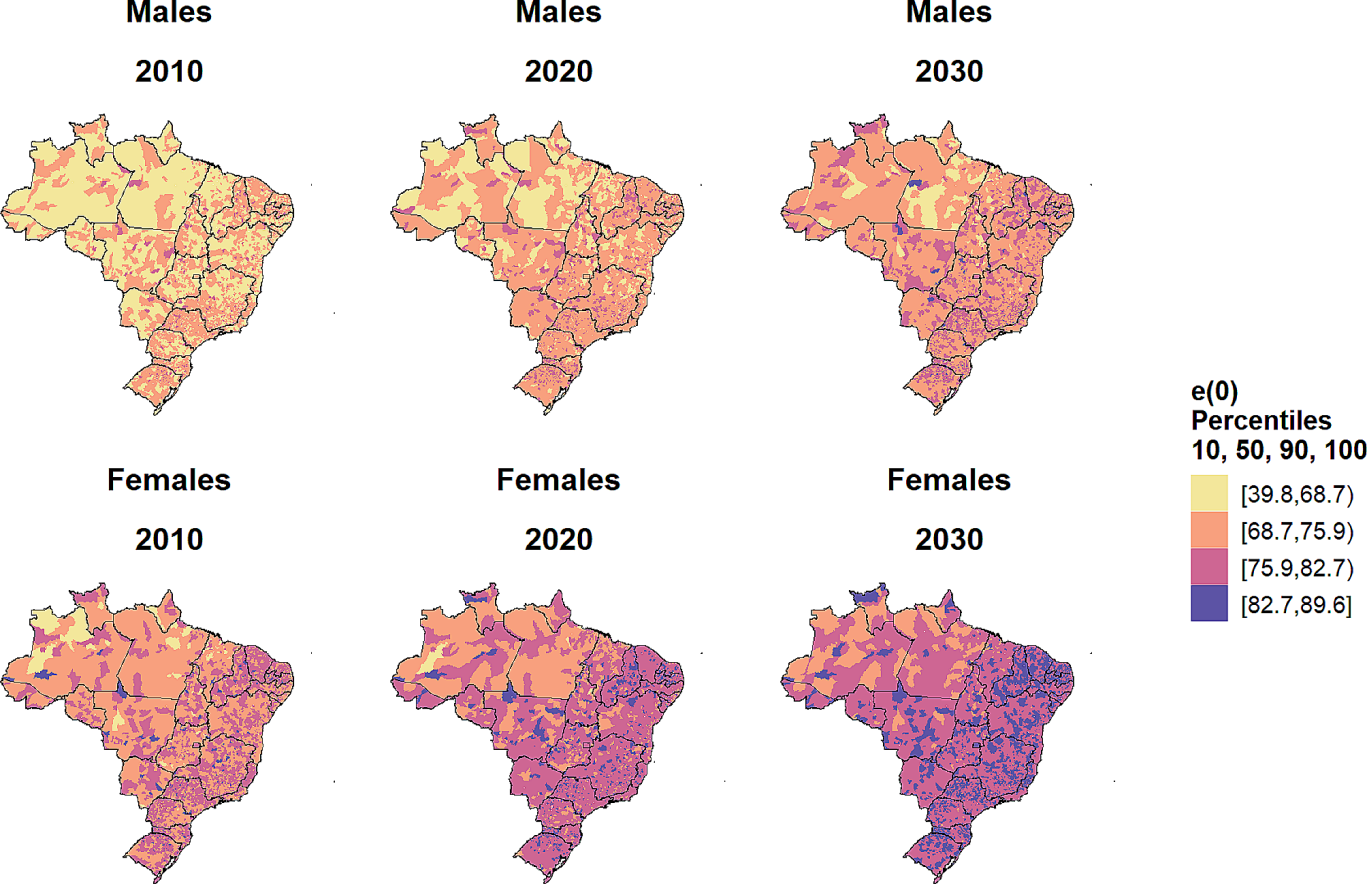
Evolution of life expectancy at birth by sex across the municipalities in Brazil (2010–2030) Source: Mortality Information System/Ministry of Health (SIM/Datasus), Brazilian Demographic Census (IBGE, 2010) and Silva [47]

Figure 6 depicts male and female estimates of life expectancy at birth in 2010, and projections for Brazilian municipalities for 2020 and 2030. To facilitate the comparison, we presented estimates divided into percentiles. The gap across locations with the highest and lowest life expectancy has narrowed over time. However, we still find several municipalities with low life expectancy levels at birth, especially among males in the north and northeast macro-regions. However, our estimates indicate a convergence process in mortality levels across macro-regions, especially for females.

**Fig. 7:**
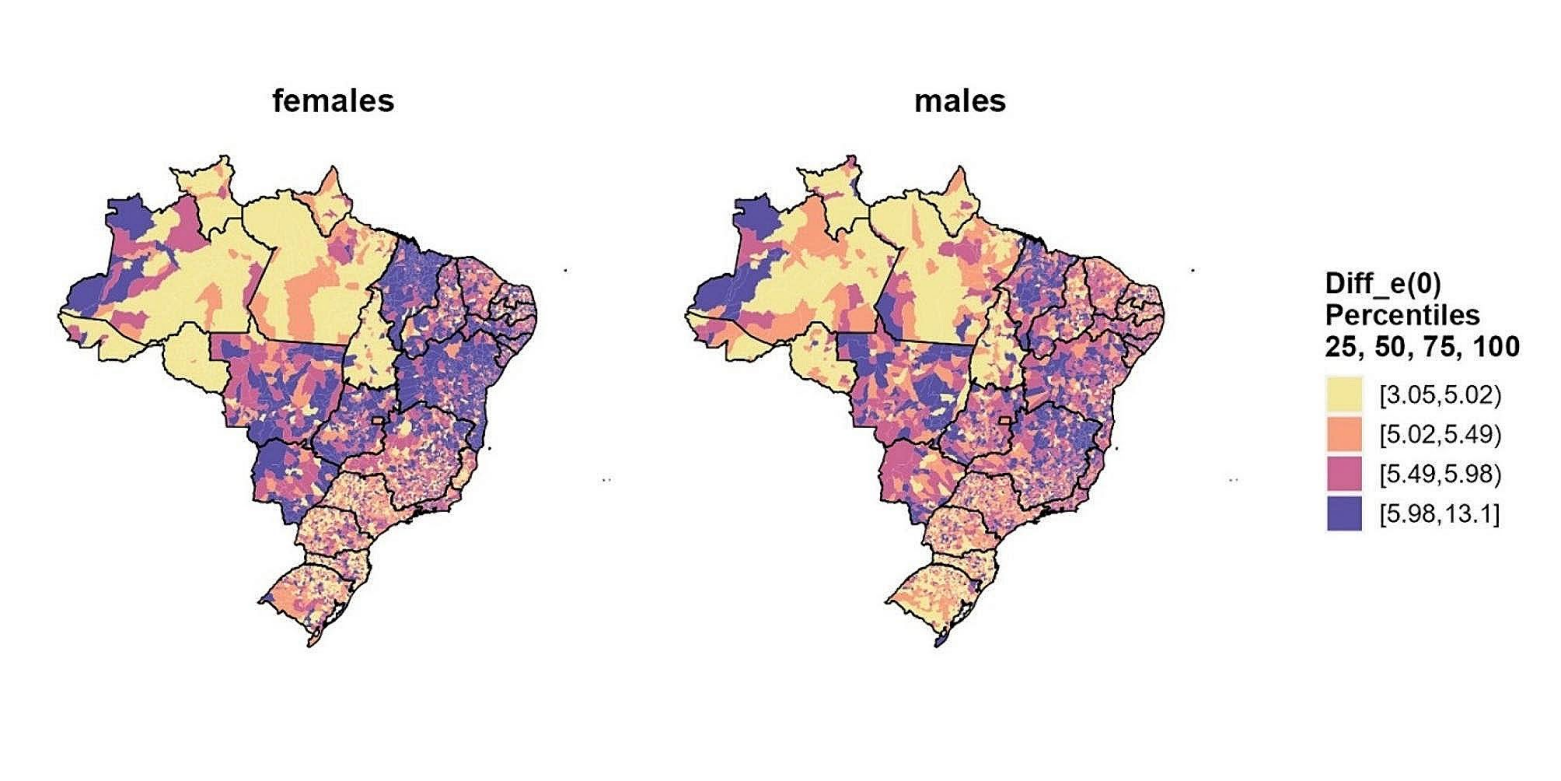
Improvements in life expectancy at birth by sex across Brazilian municipalities spanning the years from 2010 to 2030 Source: Mortality Information System/Ministry of Health (SIM/Datasus), Brazilian Demographic Census (IBGE, 2010) and Silva [47]

Figure 7 illustrates the increases in life expectancy at birth, differentiated by sex, across Brazilian municipalities between 2010 and 2030. The use of a percentile scale was employed for a more nuanced examination of differences. Municipalities situated in the Northeast and Midwest macroregions exhibit the most significant improvements in life expectancy for both sexes. Furthermore, in most municipalities, the gains in life expectancy are more pronounced for females compared to males.

Table 1 presents the evolution of the sex-based disparity in life expectancy from 2010 to 2030. Our findings indicate a reduction in the life expectancy gap between males and females in the North and Southeast regions during this period. Conversely, in other regions, particularly in the Northeast, we anticipate an increase in the sex-based life expectancy gap from 2010 to 2030. We hypothesize that this phenomenon is likely attributed to rising mortality rates from external causes among males in the Northeast region. Considering all 5.565 municipalities, our estimates indicate persistent differences in life expectancy at birth – e(0) between males and females over time, with males having the lowest values throughout the entire forecast period. Indeed, on average, female life expectancy was 6.80 years higher than that of males in 2010 and is projected to be 6.89 years higher in 2030, reflecting a positive percentage change of approximately 1.28%.

**Table 1:** Differential in Life Expectancy at Birth between sex across Brazilian macroregions from 2010 to 2030

Macroregion	Year		Percentage change
	2010	2030	
Central-West	6.16	6.37	3.38%
Northeast	7.27	7.70	5.96%
North	6.63	6.28	-5.28%
Southeast	6.78	6.59	-2.91%
South	6.67	6.75	1.18%
Brazil	6.80	6.89	1.28%

Source: Mortality Information System/Ministry of Health (SIM/Datasus), Brazilian Demographic Census (IBGE, 2010) and Silva [47]

Table 2 provides summarized results for 2010 estimates and 2030 forecast analysis. It shows the Coefficient of Variation (CV) for life expectancy at birth and Mean of Interquartile Range (IQR) for death distribution between the municipalities by sex, macro-region, and year, between 2010 and 2030. Life expectancy variability between municipalities decreases between 2010 and 2030 in all macroregions and for both sexes, meaning lower variation in terms of e(0) in 2030 compared to 2010. In addition, municipalities in the south and southeast macroregions present less variability in their mortality levels for both sexes. A probable reason is that there is only one source of uncertainty in mortality estimates in the south and southeast macroregions since the under-registration of death records in these areas was negligible in 2010.

The results on Table 2 demonstrate the average municipal IQR across macroregions by sex. A slight reduction in the IQR average across municipalities in all macroregions and for both sexes is observed, which means that, on average, all municipal age at death distribution becomes more concentrated over time. For example, considering the municipalities selected in Fig. 1, the IQR reductions varied between 0.18 (São Paulo- São Paulo) and 1.56 (Quirinópolis- Goiás) years. Slight IQR reductions may be related to high levels of premature deaths and external causes. The female IQR is lower than that for males, which means that the female distribution of age at death is more concentrated around the median age at death.

**Fig. 8:**
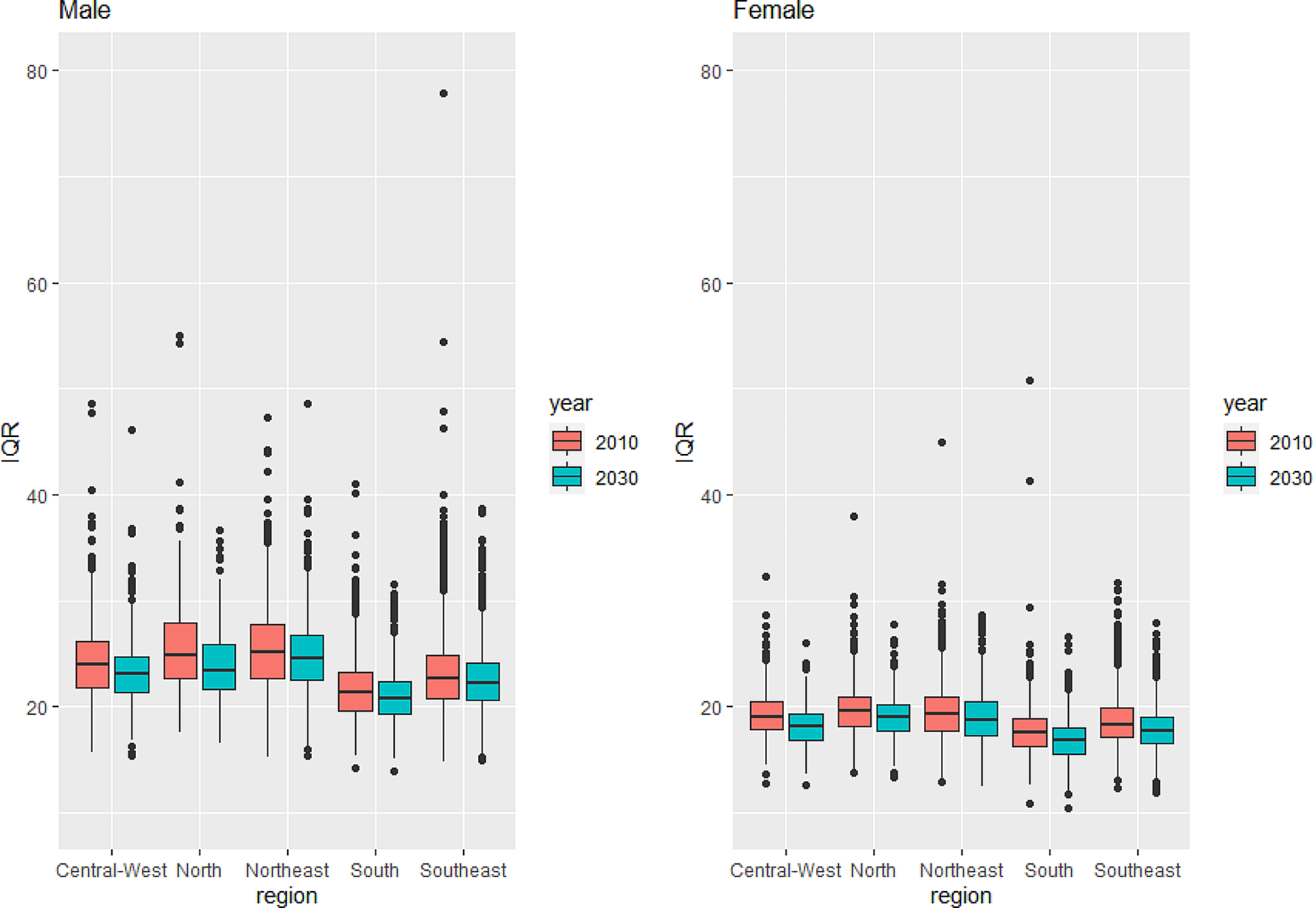
Death Interquartile Range (IQR) across the municipalities by sex and macro-region, Brazil (2010 and 2030) Source: Mortality Information System/Ministry of Health (SIM/Datasus), Brazilian Demographic Census (IBGE, 2010) and Silva [47]

As for life expectancy at birth, we observe a convergence of age at death variability between municipalities in all macroregions and by sex. That phenomenon is visually more apparent in Fig. 8. According to our projections, a reduction and convergence of municipal IQR will occur in all macroregions and both sexes. In addition, the changes in the median age at death are positively correlated with those in life expectancy at birth, and the reduction in the variability of age at death will occur in all municipalities, on average, with a shift in death distribution to older ages between 2010 and 2030. For example, considering the municipalities presented in Fig. 2, the male median age at death will increase by at least 3.73 years (in Mazagão, Amapá), while the highest increase will occur in Araçuaí-Minas Gerais (6.77 years).

**Table 2:** Life expectancy at birth coefficient of variation (CV) and mean of death interquartile range (MIQR) across the municipalities by sex, macro-region and year, Brazil (2010–2030)

Region / Measure		Male			Female		
		2010	2030 (95% IC)		2010	2030 (95% IC)	
North	CV	6.25	5.55	(5.43; 5.69)	5.48	4.91	(4.82; 5.03)
	MIQR	25.58	23.78	(23.46; 24.16)	19.86	19.06	(18.90; 19.24)
Northeast	CV	5.87	5.24	(5.19; 5.29)	4.66	4.17	(4.16; 4.18)
	MIQR	25.38	24.71	(24.67; 24.75)	19.44	18.89	(18.83; 18.96)
Southeast	CV	5.04	4.41	(4.38; 4.46)	4.23	3.83	(3.83; 3.84)
	MIQR	23.22	22.61	(22.57; 22.66)	18.69	17.92	(17.86; 17.97)
South	CV	5.09	4.53	(4.51; 4.56)	4.37	3.92	(3.91; 3.94)
	MIQR	21.7	20.95	(20.92; 20.98)	17.72	16.83	(16.73; 16.93)
Central-West	CV	5.86	5.28	(5.26; 5.32)	5.21	4.75	(4.73; 4.77)
	MIQR	24.44	23.32	(23.24; 23.40)	19.23	18.1	(17.94; 18.25)

Source: Mortality Information System/Ministry of Health (SIM/Datasus), Brazilian Demographic Census (IBGE, 2010) and Silva [47]

## Discussion

Age-specific mortality rate estimation in small areas remains a challenge for demographers, epidemiologists, public health researchers, and policymakers from low- and middle-income countries (LMICs). These estimates are central to planning public policies and input for mortality forecasts. This paper proposed a methodological approach that we demonstrated to be robust both in adjusting for the age profile and in estimating mortality rate uncertainties. We also observed that the methodology performed well for municipalities of different population sizes. These results highlighted the importance of using under-registration estimates to adjust age- and sex-specific mortality rates in small areas in Brazil or other countries with incomplete data. Based on these estimates, we used the Lee-Carter model to forecast mortality for all Brazilian municipalities between 2010 and 2030.

Municipal level mortality estimates in 2010, as expressed by life expectancy at birth, infant mortality (1q0), and adult mortality probabilities (45q15), were significantly different across and within Brazil’s macro-regions. We observed an increased mortality risk among adults in regions closer to state capitals, especially in the northeast macro-region, possibly related to mortality by external causes such as homicide and transport accidents [26, 55, 56, 60, 61]. In addition, we observed the highest infant mortality rates in the north and northeast macro-regions compared to the rest of Brazil. It is well known that infant deaths is intrinsically related to sanitary and sociodemographic conditions [59, 62], which still far from ideal in the north and northeast macro-regions. Therefore, this scenario offers a partial explanation for those findings.

Regarding the mortality trend, there was a significant difference in historical and projected mortality rates by sex, which is a pattern highlighted in previous studies [55, 56, 63]. Among male, the excess mortality in the period 2010–2030 possibly indicates that the projection is capturing the trend of increased mortality from external causes [26, 61], as discussed earlier.

In general, we observed, over time,  a shift in life table deaths to more advanced ages (> 60 years old) for both sexes. This finding may be related to a considerable decrease in mortality from preventable causes [64, 65], especially among children and young adults [56, 60]. The decline in mortality in these age groups is well documented and is forecasted to continue in the near future [59, 62]. The rectangularization of the survival curve and shift of deaths to older ages tend to occur concomitantly with decreasing overall mortality [64, 65]. This pattern may be explained by an increase in the average age at death, followed by a decrease in the dispersion of deaths around this average age of death [65–67]. The results presented in this study showed that, despite regional heterogeneity, the decline in mortality occurred across Brazil at a similar pace as in Latin America region [64, 65]. The trend towards a rectangular survival curve was more pronounced among women than men. As observed in other countries, the projections also suggest a continued decline in mortality among older age groups and an increase in the modal age at death [68, 69]. The observed decline in mortality, which is a key driver for gains in life expectancy at all ages, may be partly attributed to improvements in the Brazilian population’s social living conditions [60].

The mortality forecasts follow the expected tendency, considering the process of convergence of life expectancy and mortality levels in recent decades [55, 56]. There has been a rapid decline in mortality across macro-regions since the 1990s, particularly in child and infant mortality [56, 58]. There was also an important change in the ranking of causes of death, in particular, there has been a considerable decline in mortality by communicable, maternal, neonatal, and nutritional conditions [56, 63]. The mortality age profiles have shifted to older ages, increasing deaths by non-communicable diseases and violence [56, 58]. These tendencies are reflected in the observed and projected mortality estimates. A point that attracts attention, and deserves further studies, is the non-reduction of the mortality differential between men and women over the study period.

Our study proposed a flexible methodology that could be applied to countries with limited data at the sub-national level, since it only requires death counts by age and sex for one year, and a series of mortality estimates in larger areas. However, it has four main limitations. Firstly, we relied on the assumption that the under-registration of deaths was homogeneous across all municipalities within a micro-region. Although municipalities within the same micro-region could be similar in demographic or socioeconomic indicators, municipalities in remote areas of the north macro-region may have very particular characteristics, such as more limited access to health services. Secondly, we used Lee-Carter’s macro-region level parameters to estimate municipal level projections. There is a strong assumption here that the path of global mortality levels (*k*_*t*_) and the pace of change in age-specific mortality rates (*b*_*x*_) is similar across all municipalities within the same macro-region. Thirdly, in the Lee-Carter model, bx parameters are fixed in time. It is known that the rates of decline in mortality by age do not remain constant for long periods of analysis. More specifically, it is observed that these rates tend to be higher at younger ages, when mortality is still high. Thus, long forecasting periods may be very unstable. However, it is worth mentioning that, although there are some limitations, the Lee-Carter model is widely used, since it is parsimonious and, in combination with time series methods, can provide reliable stochastic mortality projections. Lastly, it is important to note that our projections were performed based only on 2010 baseline estimates, which is the year of the last Brazilian Census. Therefore, our projections do not take the current Covid-19 pandemic into account. There is strong evidence that life expectancy in the Brazilian states has been heavily and differently affected by the Covid-19 pandemic [40, 70]. Nevertheless, our projections are still relevant, especially considering the territorial complexity of Brazil, which comprises more than five thousand municipalities.

In the context of the Covid-19 pandemic, sex- and age-specific mortality rate projections may be important and useful information to understand the death toll of the pandemic in an excess mortality analysis. As argued by others, there are several ways to measure the impact of excess deaths on account of the pandemic [71, 72]. However, if the objective is to compare mortality levels between populations, or in the same population over time, the effects of changes in the population age structure in the indicators analyzed should be assessed [41, 73–75]. In this way, projections of sex- and age-specific mortality rates are useful to analyze the impact of the Covid-19 pandemic on life expectancy in small areas in a country such as Brazil.

## Conclusion

As argued in the literature, accurate estimation and projection of age-specific mortality rates in small areas are essential for public health, economic, and social planning [1, 2]. They may also be applied to monitor the Sustainable Development Goals [3, 4] and support the development of other small area measures [5]. However, due to (1) a limited number of events and exposure in small areas and (2) low levels of completeness of death registrations, estimating mortality rates in small areas is still challenging for demographers and epidemiologists in low- and middle-income countries, including Brazil [6–10]. In this paper, we show that combining the use of demographic and statistical methods can be efficient in estimating and forecasting mortality rates in small areas with incomplete death records and considerable heterogeneity. A key feature of this study was to estimate and forecast sex- and age-specific mortality rates for small areas in Brazil, while accounting for under-registered deaths and providing probabilistic uncertainty estimates for future rates. This contribution can be useful for public policy planning and for estimating social security costs at the local level.

As previously mentioned, our projected population trends also indicate a regional process of converging mortality levels, an increase in life expectancy, and, consequently, a rapid population aging process across regions. This demographic shift poses significant challenges to the formulation of public policies and various sectors of society. Unlike what is observed in developed countries, this phenomenon in Brazil is occurring later and faster, giving institutions less time to adapt and effectively address the needs of this aging population subgroup [76, 77].

For instance, the conditional life expectancy at age 65 + is projected to range from a minimum value of 5.70 to a maximum value of 25.56 in 2010 and to a minimum value of 7.59 to a maximum value of 28.39 in 2030. The increase in the elderly population and in longevity are expected to lead to changes in the morbidity profile of the Brazilian population. In societies with a higher proportion of elderly individuals, the prevalence of chronic diseases is typically elevated, thereby increasing the demand for healthcare services [78]. This shift in the epidemiological and morbidity profile will have implications for healthcare spending, necessitating a reorganization of the healthcare system to provide appropriate care for the growing elderly population. In the case of chronic conditions, treatment generally requires more complex care, often involving advanced technology, which will significantly impact healthcare expenses [79].

The results redirect to a broader research agenda, including a better understanding of Brazil’s social and economic determinants of mortality inequalities [80, 81], more in-depth studies on data quality in the states and smaller areas, and methodological and substantive studies on mortality inequalities by sex. The study also highlights the importance of strengthening the quality of Civil Registration and Vital Statistics systems, as they are central to public health planning and to tracking mortality population patterns. Finally, we suggest future adaptations of our methodology to estimate the excess of deaths due to the Covid-19 pandemic, and to analyze the pandemic’s impact on the future path of life expectancy in small Brazilian areas.

### Electronic Supplementary Material

Below is the link to the electronic supplementary material.

Supplementary Material 1

## Data Availability

The R codes, datasets and supplementary information supporting the conclusions of this article are available in the https://github.com/blanza/paperPHM. The data used are publicly available at: a) Instituto Brasileiro de Geografia e Estatística (IBGE): www.ibge.gov.br. b) Minister of Healthy/Mortality Information System (DATASUS): https://datasus.saude.gov.br.

## Abbreviations

CV: Coefficient of Variation
IBGE: Brazilian Institute of Geography and Statistics
IQR: Interquartile Range
SIM: Mortality Information System
SVD: Singular Value-Decomposition
