# Supplementary material for: Estimation and probabilistic projection of age- and sex-specific mortality rates across Brazilian municipalities between 2010 and 2030
Source: Popul Health Metr. 2024 May 27;22:9. doi: 10.1186/s12963-024-00329-x (PMC11129360; doi:10.1186/s12963-024-00329-x)
Supplement: Supplementary file 1 — Supplementary Material 1 [file 12963_2024_329_MOESM1_ESM.docx]

**Supplementary information**

Additional Figure S1 Completeness of death estimates across micro-regions by sex, age interval, and macro-region, Brazil (2010)


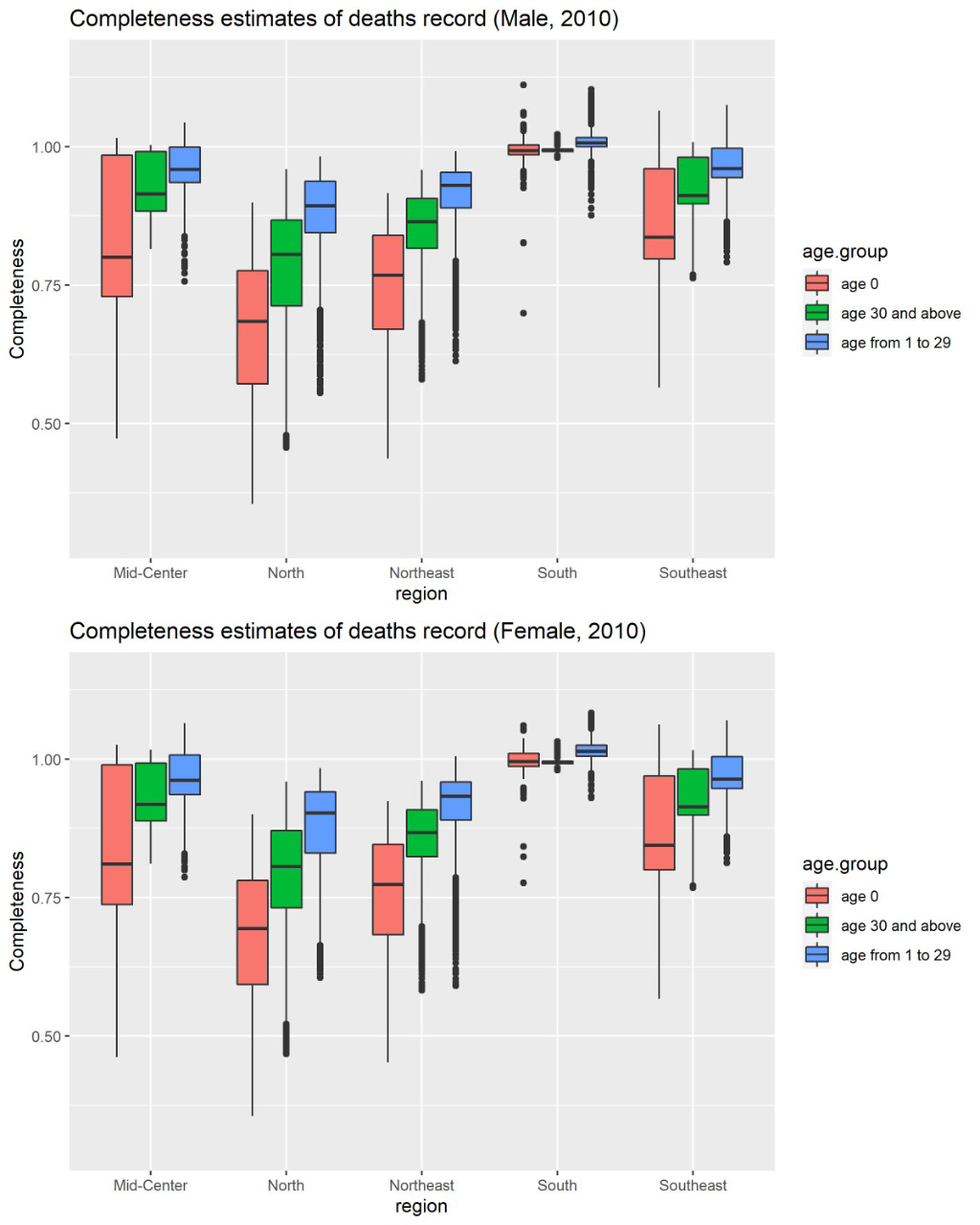


Source: Mortality Information System/Ministry of Health (SIM/Datasus), Brazilian Demographic Census (IBGE, 2010).

Additional Figure S2 Lee-Carter forecasted life expectancy at birth, selected municipalities of Brazil (2010: 2030)


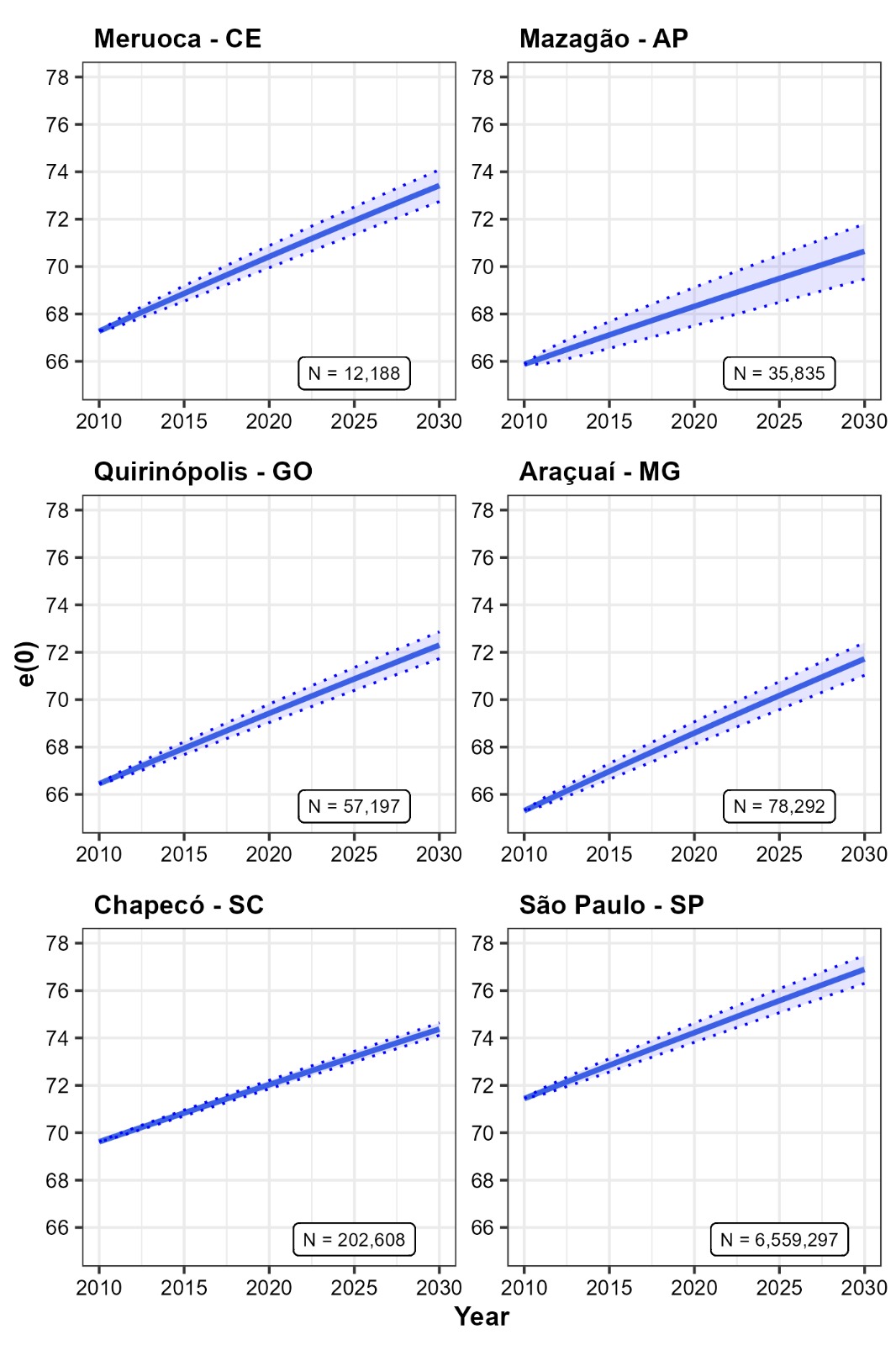


Source: Mortality Information System/Ministry of Health (SIM/Datasus), Brazilian Demographic Census (IBGE, 2010).
